# Use of substructure-specific carbohydrate binding modules to track changes in cellulose accessibility and surface morphology during the amorphogenesis step of enzymatic hydrolysis

**DOI:** 10.1186/1754-6834-5-51

**Published:** 2012-07-24

**Authors:** Keith Gourlay, Valdeir Arantes, Jack N Saddler

**Affiliations:** 1 Forest Products Biotechnology/Bioenergy Group, Department of Wood Science, Faculty of Forestry, University of British Columbia, 2424 Main Mall, Vancouver, BC, V6T 1Z4, Canada

**Keywords:** Amorphogenesis, Cellulose Accessibility, Swollenin, Cellulose Disruption, Carbohydrate Binding Modules, Enzymatic Hydrolysis, Biofuels

## Abstract

**Background:**

Cellulose amorphogenesis, described as the non-hydrolytic “opening up” or disruption of a cellulosic substrate, is becoming increasingly recognized as one of the key steps in the enzymatic deconstruction of cellulosic biomass when used as a feedstock for fuels and chemicals production. Although this process is thought to play a major role in facilitating hydrolysis, the lack of quantitative techniques capable of accurately describing the molecular-level changes occurring in the substrate during amorphogenesis has hindered our understanding of this process.

**Results:**

In this work, techniques for measuring changes in cellulose accessibility are reviewed and a new quantitative assay method is described. Carbohydrate binding modules (CBMs) with specific affinities for crystalline (CBM2a) or amorphous (CBM44) cellulose were used to track specific changes in the surface morphology of cotton fibres during amorphogenesis. The extents of phosphoric acid-induced and Swollenin-induced changes to cellulose accessibility were successfully quantified using this technique.

**Conclusions:**

The adsorption of substructure-specific CBMs can be used to accurately quantify the extent of changes to cellulose accessibility induced by non-hydrolytic disruptive proteins. The technique provided a quick, accurate and quantitative measure of the accessibility of cellulosic substrates. Expanding the range of CBMs used for adsorption studies to include those specific for such compounds as xylan or mannan should also allow for the accurate quantitative tracking of the accessibility of these and other polymers within the lignocellulosic biomass matrix.

## Background

Over the past 50 years a considerable amount of research has been dedicated to determining the roles of the hydrolytic proteins involved in the solubilisation and depolymerisation of the carbohydrates within the lignocellulosic biomass matrix [1-3]. In its native state, cellulose chains typically exist in tightly packed bundles encased within a complex sheath of hemicelluloses and lignin [4-6]. In order for cellulases to hydrolyze the glycosidic linkages within these chains, they must first be able to diffuse into this dense, heterogeneous matrix and access the cellulose [7].

It is becoming increasingly apparent that enzymatic deconstruction of cellulose occurs through two distinct steps. First, an initial disruption of the substrate, the so-called “cellulose amorphogenesis” phase, is thought to be mediated at least in part by non-hydrolytic disruptive proteins [8]. This step is required to enhance the accessibility of the cellulose to the cellulase enzyme mixture [8] while, in the subsequent step, the cellulase enzymes diffuse into and hydrolyze the cellulose. Although the basic functions and mechanisms of the major hydrolytic enzymes have been studied extensively, little is known about the role of the non-hydrolytic proteins that have been suggested to be involved in disrupting the substrate prior to hydrolysis [8]. By developing a better understanding of the role that non-hydrolytic proteins play in the disruption of lignocellulosic materials, it should be possible to design more efficient enzyme preparations, thereby bringing us one step closer to achieving an effective, enzyme/sugar-based biorefinery [5,9-11].

### Protein-induced amorphogenesis

Several cellulolytic organisms have been shown to produce non-hydrolytic proteins capable of disrupting cellulosic and lignocellulosic substrates (Reviewed in [8]). While the exact mechanisms by which these proteins disrupt the substrate have yet to be fully resolved, qualitative and semi-quantitative observations have suggested that this disruption can be manifested as a delamination, fibrillation, swelling, loosening, roughening, pitting, weakening, or decrystallization of cellulosic and lignocellulosic substrates. The term “amorphogenesis” has been suggested as a way of describing any combination of these phenomena induced by non-hydrolytic proteins [8].

**Figure 1:**
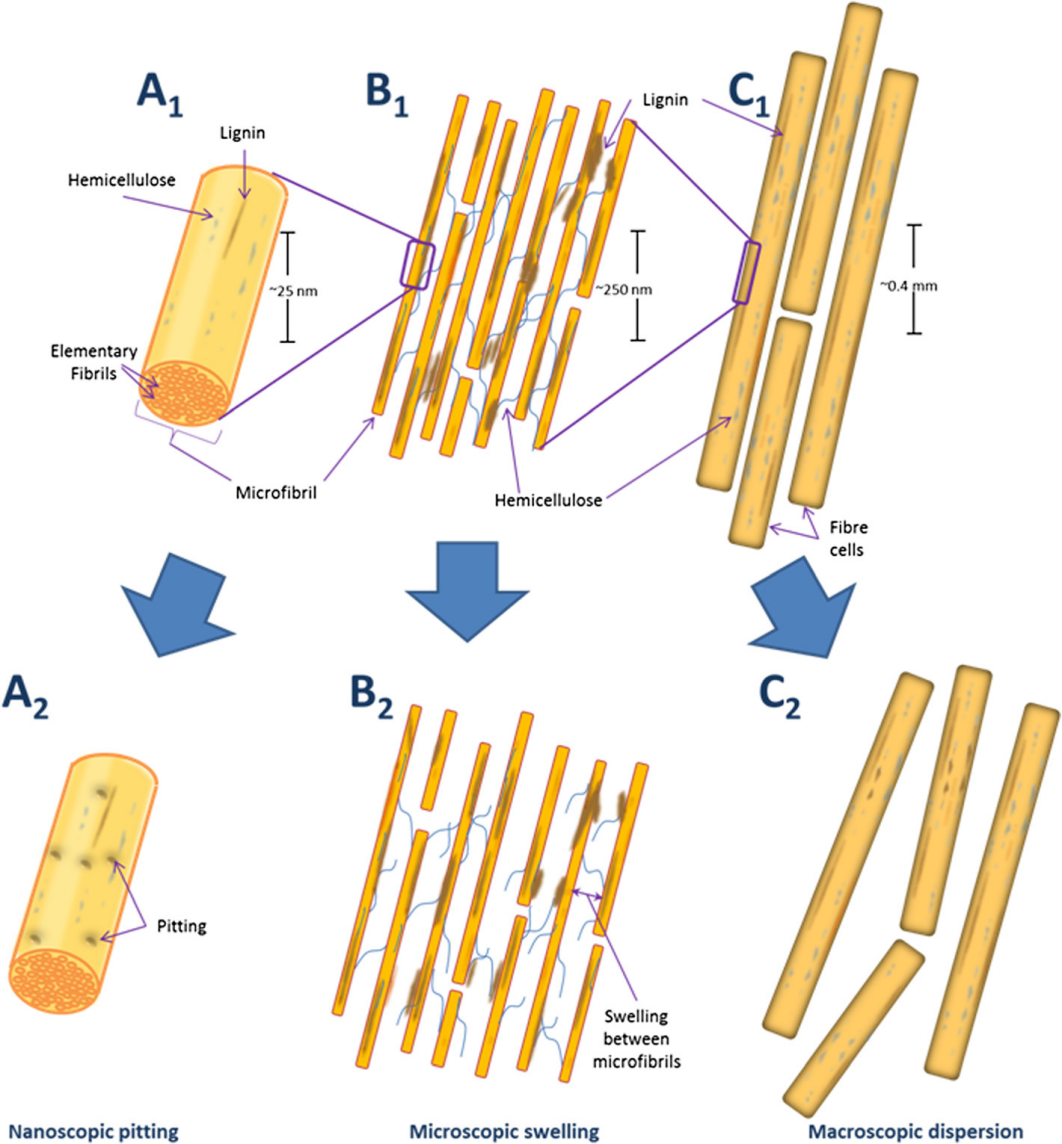
**Simplified schematic representation of amorphogenesis occurring at three levels of biomass organization.** Native plant material at the nanoscopic (**A**_**1**_), microscopic (**B**_**1**_) and macroscopic (**C**_**1**_) levels of organization. After amorphogenesis induced by non-hydrolytic disruptive proteins, nanoscopic pitting (**A**_**2**_), microscopic swelling/roughening (**B**_**2**_), and macroscopicfibre dispersion (**C**_**2**_) is observed. These effects occur without significant release of sugars from the substrate.

In previous work we [12,13] and other workers [14] have suggested that accessibility challenges at the macroscopic (fibre), microscopic (fibril) and nanoscopic (microfibril) level restrict effective enzymatic hydrolysis. Interestingly, non-hydrolytic disruptive proteins have been shown to disrupt the substrate at each of these three organizational levels. Thus it is likely that these proteins play a key role in enhancing the effectiveness of enzymatic hydrolysis. For example, amorphogenesis induced by non-hydrolytic disruptive proteins has been observed at, 1) the macroscopic level through the dispersion of adjacent fibres [15-17], 2) the microscopic level through the loosening/roughening/swelling of plant cell walls [16-27], and 3) at the nanoscopic level through the pitting of microfibrils and cellulose decrystallization (Figure 1) [17,23,26,28-30].

Recently, it has been suggested [8] that non-hydrolytic disruptive proteins can be categorized into two distinct groups. Those with an as yet unknown catalytic mechanism such as Swollenin [16,17], Loosenin [27], Expansins[21,31] and several Family 1 and Family 2 CBMs[18,19,23,30] and those thought to act through a direct oxidative catalytic mechanism, such as GH61 [32-36] and Family 33 CBMs [37,38] (Table 1). These two groups of proteins are thought to act in distinctly different ways on the substrate. For example, several of the proteins with uncharacterized catalytic function, such as Swollenin, Expansins and Loosenin, are thought to promote amorphogenesis through disruption of the hydrogen bonding network of the substrate (i.e. without direct cleavage of the carbohydrate chains) [16,39]. In contrast, the oxidative proteins that act on the substrate do so through a direct catalytic oxidative mechanism, where radical species are generated in close proximity to the cellulose surface, resulting in the direct oxidative cleavage of the cellulose chains [33-38].

**Table 1:** Non-hydrolytic disruptive proteins and their effects on biomass

**Proteins with unknown catalytic mechanism**	**Putative function**	**References**
Family 1 and 2 CBMs	Fibre pitting/roughening, small particle release	[18,19,23,30]
Swollenin, Loosenin	Fibre swelling, microfibril dispersion, dispersion of cellulose aggregates	[16,17,27]
Expansins	Loosening of plant cell walls, solubilization of oligomeric sugars	[21,31,39]
Expansin-like proteins	Loosening of filter paper, dispersion of cellulose aggregates	[25,78]
Fibril Forming Protein	Fibril release from filter paper	[15]
**Proteins with putative oxidative catalytic mechanism**	**Putative function**	**References**
GH61	Oxidative cleavage of crystalline cellulose	[33-36]
CBM33	Oxidative cleavage of crystalline cellulose	[37,38]

The non-hydrolytic proteins capable of promoting amorphogenesis of cellulosic and/or lignocellulosic biomass identified to date. Amorphogenesis induced by these proteins has been shown to manifest as a range of specific disruptive effects.

A common theme between these two groups of proteins appears to be the release of soluble oligomers from the substrate. Specifically, Beta-Expansins have been shown to solubilize both hemicellulose and pectin from native maize silk cell walls [31], while the oxidative proteins GH61 and CBM33 promote the release of soluble cello-oligosaccharides from model cellulosic substrates [33,37,38]. Further evidence supporting the role of non-hydrolytic proteins in cellulose amorphogenesis comes from reports that disruptive proteins with unknown catalytic mechanisms appear to enhance the enzymatic hydrolysis of native and pretreated substrates [17,20,23-25,27,29,40-43]. In addition to these proteins, hydrolysis enhancing activity has also been observed for the oxidative disruptive proteins. In particular, GH61 has been shown to significantly reduce the total protein required to achieve 70-80% hydrolysis yields of a pretreated corn stover substrate [32]. These results suggest that non-hydrolytic proteins can act within a similar time scale to that of enzymatic hydrolysis and that they are capable of promoting further amorphogenesis within a substrate that has already been disrupted by a physicochemical pretreatment.

These earlier observations encouraged various research groups to try to develop a better understanding of the role that non-hydrolytic proteins might play in promoting the amorphogenesis of lignocellulosic substrates, with the expectation that further elucidation of their action might help in the development of more efficient commercial enzyme preparations. However, one of the key limitations in better characterizing the non-hydrolytic proteins involved in biomass deconstruction has been the lack of simple quantitative techniques for measuring changes in cellulose accessibility. Various techniques have been used, with mixed success, to try to quantify changes in cellulose accessibility and their relative merits are discussed below. We also describe a novel technique, using the adsorption of substructure-specific CBMs over short time scales (<30 minutes) to quantify cellulose accessibility [44] and to accurately quantify the degree of amorphogenesis induced by non-hydrolytic disruptive proteins.

### Techniques for measuring amorphogenesis

Although a range of quantitative techniques can be used to measure the second (hydrolytic) step of cellulose deconstruction [45-47], few if any current techniques are able to provide an accurate quantitative measure of how the enzyme mixture might increase cellulose accessibility or what has been termed the amorphogenesis step of enzyme mediated cellulose hydrolysis. This is in part due to the nature of the end product of each step. After enzymatic hydrolysis, the end products (soluble sugars) are readily quantified by high performance liquid chromatography or by using colorimetric techniques such as the dinitrosalicylic acid [45] or glucose oxidase assays [46,47]. In contrast, the end products of the amorphogenesis step are challenging to describe, let alone quantify [17].

A major challenge in trying to quantify amorphogenesis is that the method or technique has to be versatile enough to accurately quantify a range of effects occurring at different levels of biomass organization, varying in scale by several orders of magnitude (from the microfibril, with typical diameters of 3–5 nm and lengths of 100 s to 1000 s of nm, up to the whole fibre, with diameters of 5–50 μm and lengths of 1–4 mm) [5,48,49]. This point was recently highlighted by Jäger *et al.* (2011), who noted that no single technique has the capacity to simultaneously quantify the effects occurring at multiple levels of cell wall organization [17]. As a result, previous attempts to measure these effects have typically made use of a suite of complementary qualitative and semi-quantitative techniques [15-20,22-30].

The most widely used methods employed to try to confirm disruptive protein mediated amorphogenesis of biomass typically involve the application of qualitative microscopic techniques. Light microscopy has been used to try to assess the macroscopic dispersion of Valonia cell walls and microscopic swelling of cotton fibres induced by the fungal disruptive protein Swollenin [16]. Scanning electron microscopy (SEM) has also been used to show the microscopic roughening of cotton fibres by Swollenin [17] and by the CBMs from the bacteria *Cellulomonas fimi* and *Clostridium cellulovorans* and the fungus *Trichoderma reesei *[19,22,26]. Additionally, atomic force microscopy (AFM) has been used to show nanoscopic pitting of cotton microfibrils induced by CBM1 from *Trichoderma pseudokoningii* S-38 [28]. However, while these techniques have provided useful qualitative information on the effects of disruptive proteins on model cellulosic substrates, attempts to quantify these effects have so far been limited to either monitoring changes in crystallinity [17,23,26,29,30], measuring the release of small particles [19,20,23] or by indirectly quantifying amorphogenesis by measuring changes in the ease of hydrolyzability of the substrate induced by these proteins [17,24,27,42,43]. The various methods previously used to try and quantify amorphogenesis are discussed below.

### Crystallinity

There are conflicting opinions on how influential cellulose crystallinity is on limiting enzymatic hydrolysis and the effect that amorphogenesis-inducing proteins might have on enhancing cellulose hydrolysis. Earlier work using Fourier-transform infrared spectroscopy to assess the influence of CBM1 from *T. pseudokoningii* S-38 on cotton fibre deconstruction claimed that the addition of CBM1 helped reduce substrate crystallinity [30], while the highly similar CBM1 from *T. reesei* when added to Whatman CF11 cellulose fibres did not appear to result in any decrease in substrate crystallinity when measured using X-ray diffraction [22]. In contrast, the addition of bacterial derived CBM3a from *C. cellulovorans* reduced the crystallinity of cotton fibres when assessed by both Fourier-transform infrared spectroscopy and X-ray diffraction [26] while a recombinant Swollenin, Swo2 from *T. pseudokoningii* S-38 apparently caused an increase in the crystallinity of Avicel PH-101 [50]. Conversely, the application of a recombinant Swollenin from *T. reesei* resulted in a decrease in the crystallinity of filter paper, alpha-cellulose and Avicel when measured by powder X-ray diffraction [17].

Although these non-uniform observations might suggest that different combinations of disruptive protein and substrate result in different changes in crystallinity, it is more likely that these varied results are due to issues with the methods used to measure crystallinity. These issues include the interpretation of results from the different methods for measuring crystallinity and the applicability of extrapolating crystallinity measurements to suggest the degree of amorphogenesis. In earlier work [8] it was suggested that amorphogenesis primarily resulted from substrate changes such as cellulose delamination or fibrillation, where relatively large, intact fragments, still containing crystalline regions, are released from the bulk of the substrate. Pinto *et al.* (2004) have also suggested that non-hydrolytic disruptive proteins could increase the accessibility of cellulosic substrates without affecting the crystallinity. These workers reported no decrease in the crystallinity of cotton fibres after treatment with a non-hydrolytic disruptive protein, while observing a roughening of the cotton fibres as visualized by SEM [22]. A possible parallel mechanism is that the increase in cellulose accessibility could result from the swelling or loosening of the interactions between microfibrils, resulting in the overall weakening of the cell wall while leaving the crystalline cores of the microfibrils relatively untouched. Thus it is possible that any changes in the crystallinity of the substrate would only occur as a secondary effect of the more general process of amorphogenesis.

### Particle release and size reduction

Earlier work claiming that certain CBMs could induce amorphogenesis was carried out using CBM2a from *C. fimi *where their addition to cotton fibres resulted in the release of small particles without a concomitant release of reducing sugars [19,20]. However, although this technique could semi-quantitatively describe the release of particles from the substrate, it provided no characterization of the residual substrate.

A related approach to measuring fragmentation was employed by two independent groups to study the effects of the Swollenin proteins AfSwo1 and TasSwo1 from *Aspergillus fumigatus* and *Trichoderma asperellum*, respectively, on Avicel PH-101 [24,43]. These researchers used light microscopy to try to quantify size reduction in Avicel particles after incubation with Swollenins, with Chen *et al.* (2010) using image-analysis software to demonstrate an almost 2-fold reduction in the size of the Avicel particles [24].

### Hydrolysis enhancement

Several independent research groups have demonstrated enhanced enzymatic hydrolysis of cellulosic and lignocellulosic substrates after treatment with non-hydrolytic disruptive proteins [17,20,23-25,27,29,32,40-43]. Although this work collectively suggests that these disruptive proteins are indeed capable of enhancing the hydrolyzability of model and native cellulosic substrates, the “degree of hydrolysis enhancement” method is an indirect approach to try to quantify cellulose amorphogenesis. For example, it is possible that some of the enhancement of hydrolysis observed after addition of disruptive proteins could be due to these proteins binding to and blocking lignin, thereby preventing non-productive adsorption of cellulases to the lignin [51,52], rather than through a direct “disruptive” effect. Additionally, the somewhat contradictory results observed when similar Family 2 CBMs from Cel6A and Xyn10A from *C. fimi *were used to test for hydrolysis enhancement places further doubt on the suitability of using enhancement of substrate hydrolyzability as a tool for accurately quantifying amorphogenesis [20,53].

More recent attempts at quantifying cellulose disruption induced by non-hydrolytic proteins have exploited the synergism observed between these proteins and endoglucanases specific for amorphous regions of cellulose [27,43]. This method uses quantification of sugar released by endoglucanases from a disrupted cellulosic substrate as an indirect measure of the degree of amorphogenesis of the substrate induced by the non-hydrolytic disruptive proteins. Although this technique has been successfully applied to “semi-quantitatively” measure the disruptive effects of Swollenin on Avicel [43] and Loosenin on cotton fibres [27], one drawback of this technique is the specificity of the endoglucanases for amorphous cellulose [27,43]. As the endoglucanases employed are specific for amorphous cellulose this approach will only work well if the amorphogenesis step results in a simple decrystalization of cellulose. However, it will not measure the other possible influences of enhanced cellulose accessibility such as the splitting, delaminating or loosening of the cellulose, which could occur without any significant changes in the crystallinity of the cellulose.

### Other potential methods for quantifying amorphogenesis

While each of the putative indications of amorphogenesis, such as delamination, fibrillation, swelling, loosening, roughening, pitting, weakening or decrystallization of the substrate can all be thought of as distinct processes, if they play a role in amorphogenesis we should be able to increase access of the enzymes to the cellulose without a significant increase in the release of reducing sugars. Several groups have tried to develop methods of accurately quantifying changes in the accessibility of lignocellulosic substrates, with many of these techniques modified from traditional pulp and paper procedures [54]. Although these techniques have previously only been used to assess the overall accessibility of the substrate, including accessibility to the lignin and hemicelluloses as well as the cellulose, some of these techniques also have potential for quantifying the amorphogenesis step.

Techniques with potential for quantifying cellulose accessibility and the amorphogenesis step include measuring the water retention value, mean fibre size, nitrogen adsorption capacity and mercury porosimetry of the cellulosic substrate. Or, performing techniques such as solute exclusion, differential scanning calorimetry, time-domain nuclear magnetic resonance, Simons’ Staining and protein adsorption (Reviewed in [54]). One of the benefits of these modified pulp and paper techniques is that they can monitor changes in the cellulose accessibility at the macroscopic, microscopic and nanoscopic levels. For example, fibre size measurements give an indication of macroscopic changes in accessibility while techniques such as mercury porosimetry and solute exclusion can be used to quantify changes in pore size distribution at the microscopic level [54]. The ability to monitor changes in the substrate at the macroscopic, microscopic and nanoscopic levels would likely be of great value when quantifying cellulose amorphogenesis, as this phenomenon could occur at each of these levels.

Two techniques with potential for quantifying changes in cellulose accessibility include differential scanning calorimetry [55,56] and time-domain nuclear magnetic resonance [57], which were developed to assess pore volume and distribution within cellulosic and lignocellulosic materials. These techniques can be used to differentiate between primary bound water (water directly bound to the substrate surface with severe restrictions on conformational changes), secondary bound water (water in close proximity to the substrate where hydrogen bonding networks propagating from polar groups at the substrate surface and capillary forces place some degree of conformational constraints on the water molecules) and the free/bulk water (water distal to the substrate, which is not conformationally constrained) [57]. Thus a quantitative measurement of the total water-accessible surface area of the substrate can be obtained while providing an insight into the overall pore size distribution. Although the differential scanning calorimetry and time-domain nuclear magnetic resonance methods have not yet been used to quantify overall cellulose accessibility, it is likely that these techniques could be adapted to provide some quantitative information on the extent and mechanism of disruption induced by non-hydrolytic disruptive proteins.

An alternative technique with potential for measuring cellulose accessibility involves the use of protein adsorption to try to quantify the amount of cellulose in the substrate that is accessible to enzymes [7,12,44,58]. This technique makes use of the cellulases themselves as accessibility probes, where either a mixture of cellulases or monocomponent cellulases are incubated with the substrate followed by quantifying the amount of protein that is adsorbed to the substrate. While this technique might provide a good indication of the amount of accessible surface area of the substrate, there are two key problems to be overcome when using this approach. First, unless the adsorption study is carried out at low temperatures (which will not be representative of hydrolysis reaction conditions!), the cellulases will hydrolyze the substrate, thereby changing the substrate accessibility during the course of the assay. Secondly, cellulases are known to adsorb unproductively to lignin, which restricts the accurate quantification of cellulose accessibility [51,59].

Other recent attempts at using cellulases to quantify accessibility have involved the production of a fluorescently-tagged non-hydrolytic CBM to enhance the accuracy and sensitivity of the protein adsorption technique [44]. This technique makes use of BSA blocking to overcome problems with lignin-binding [60]. However, one potential drawback is that different CBMs recognize different substructures within the substrate [61,62]. Thus, it is possible that a probe making use of a single CBM might primarily be quantifying the accessibility of a specific cellulosic substructure, rather than the overall accessibility of the cellulose. An alternative strategy might be to use cellulase inhibitors that limit or prevent substrate hydrolysis during protein adsorption experiments at more typical substrate hydrolysis temperatures (i.e. 50°C). However, while inhibitors such as hexachloropalladate have been shown to inhibit Cel7a from *T. reesei*, it has only a limited effect on most of the other enzymes present in commercial cellulase preparations [63,64]. Thus the inhibition approach would only work when using monocomponent cellulases for the adsorption studies.

Another procedure for measuring accessibility is the Simons’ stain method [65]. This technique involves quantifying the adsorption of an anionic direct dye which has a higher affinity for cellulose than to lignin and hemicellulose [65,66]. The amount of dye bound to the cellulosic substrate gives a good indication of the total amount of cellulose accessible to cellulase within the substrate [7].

Overall, these methods have proven to be useful in quantifying some of the changes that occur in the substrate during enzymatic hydrolysis. However, there are several drawbacks to using these techniques to measure changes in cellulose accessibility. For example, the water retention value is known to be insensitive to small changes in fibre characteristics and would not be able to detect changes in the cellulosic component during or after amorphogenesis. Another major drawback of many of these techniques is that they measure the overall accessibility of the substrate, including the amount of accessible lignin and hemicellulose, not just the cellulose. This restricts techniques such as nitrogen adsorption, solute exclusion, differential scanning calorimetry and time-domain nuclear magnetic resonance from being used to assess changes in the specific amount of accessible cellulose within the substrate during or after the amorphogenesis process. Finally, some of these techniques are labour intensive, such as the solute exclusion technique, or require the use of toxic heavy metals, as in determining mercury porosimetry.

To date, no single technique has provided an accurate quantitative measure of the changes occurring within the cellulosic substrate during amorphogenesis. In the work described below we describe a novel method where cellulose substructure-specific CBMs have been successfully used to quantify amorphogenesis by determining changes in the accessibility and surface morphology of cellulose before and after treatment with amorphogenesis-inducing agents.

## Results and discussion

**Figure 2:**
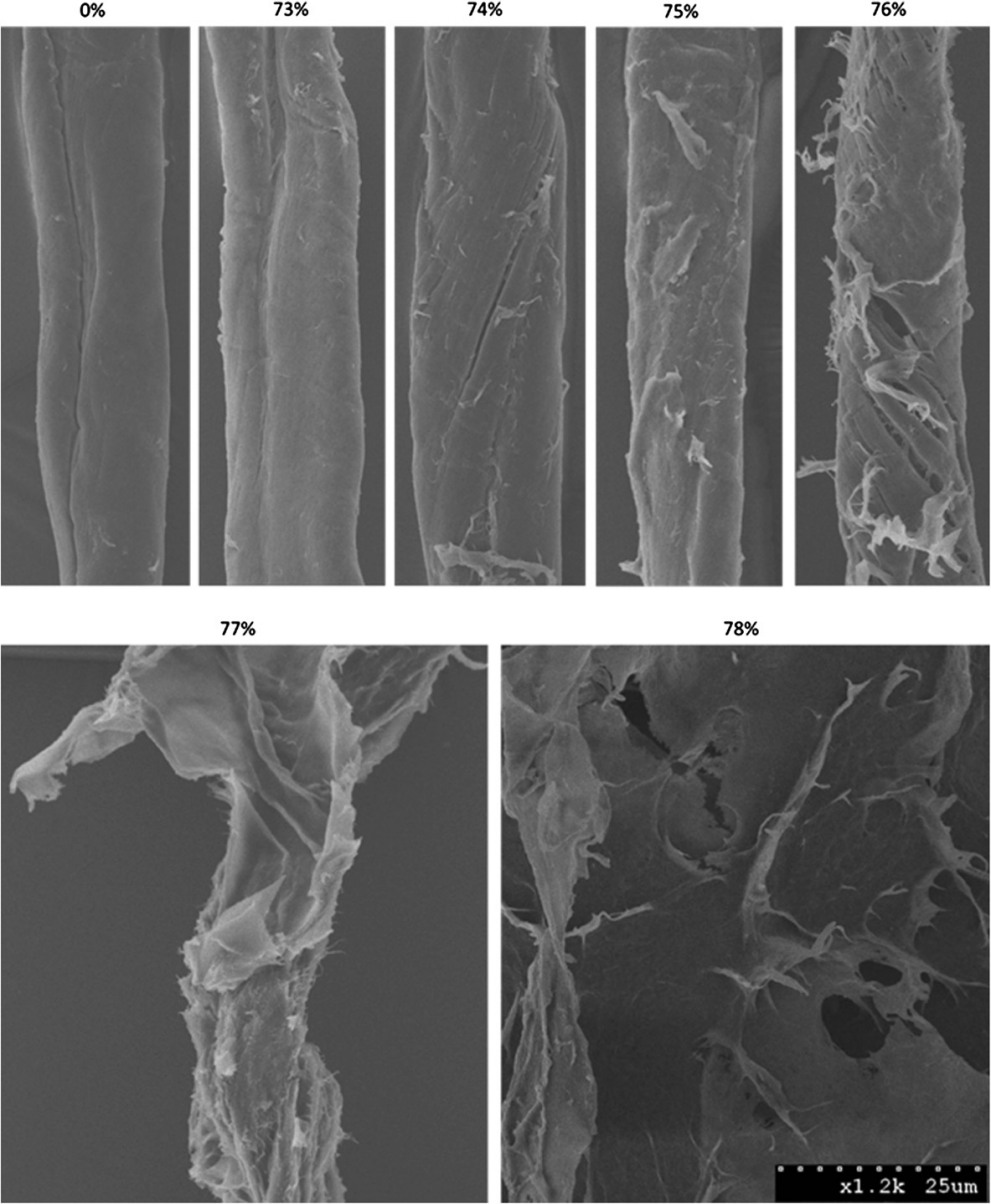
**SEM images of cotton fibres disrupted by phosphoric acid treatments.** SEM micrographs of cotton fibres after treatment with a range of *o*-phosphoric acid concentrations. After control treatment (nanopure water, 0% (w/w) acid), cotton fibres appear smooth, with few surface features. As the acid concentration was increased to near the point of cellulose dissolution (~73%-78%), manifestations of amorphogenesis begin to appear at the surface of the cotton fibres. At 74% phosphoric acid, initial signs of splitting, roughening, fibrillation and peeling/delamination of the fibres appear. As the acid concentration is increased from 74% to 76%, these effects become more pronounced. At 77%, the fibre structure has been almost completely destroyed, with large portions of the outer layer of the fibre appearing to peel off, revealing a rough, fibrillated underlying structure. After treatment with 78% phosphoric acid no fibre structure remains. All cellulose present appears to have been dissolved and reprecipitated into amorphous cellulosic ‘mats’. All images were taken at x1200 magnification and each image depicts a representative fibre for the indicated acid concentration.

**Figure 3:**
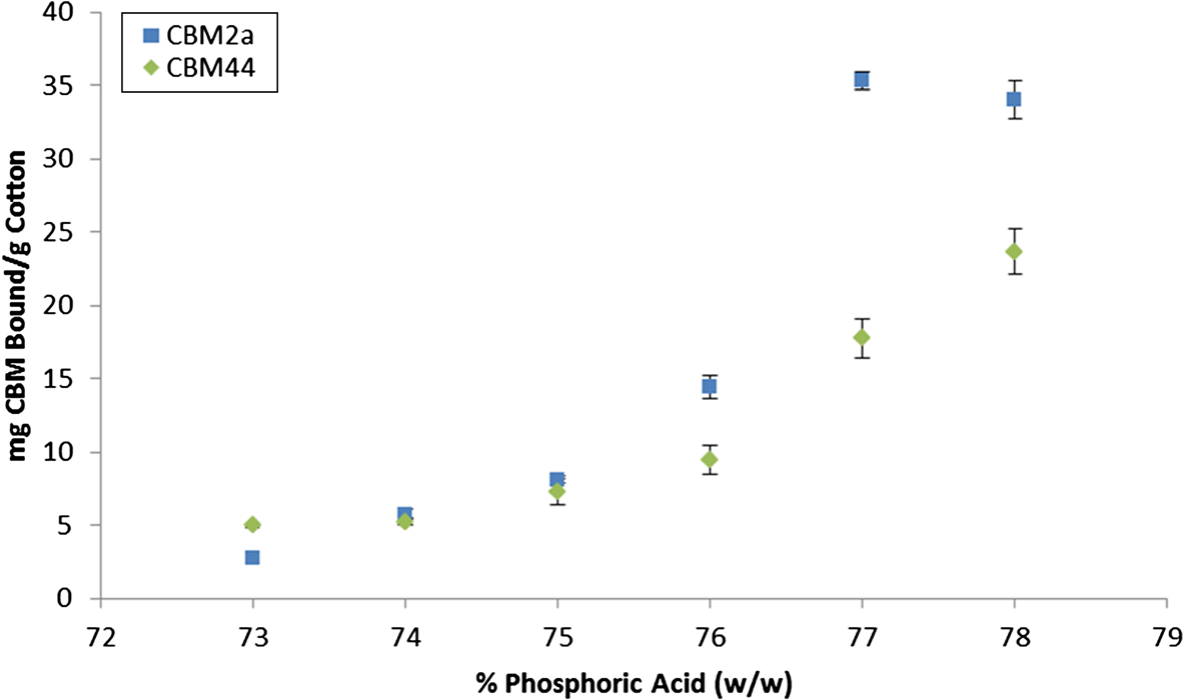
**Adsorption of CBMs to variably-disrupted cotton fibres.** Adsorption of crystalline cellulose-specific CBM2a and amorphous cellulose-specific CBM44 to cotton fibres treated with a range of *o*-phosphoric acid concentrations. Experiments were run in triplicate and error bars represent one standard deviation from the mean.

As protein-mediated amorphogenesis is still an evolving concept, we initially assessed the sensitivity and reproducibility of a CBM-mediated method for quantifying changes in cellulose accessibility by using concentrated phosphoric acid to disrupt cotton fibres to varying degrees of disassociation [67]. The use of harsh acid treatments was intended to provide an exaggerated range of disrupted substrates, and was not intended to be representative of milder, biological treatments. The disruptive effect of the acid was initially qualitatively assessed using SEM (Figure 2) followed by a quantitative assessment where the adsorption of each of the substructure-specific CBMs [61] was determined (Figure 3).

The CBM2a and CBM44 were used to specifically detect the crystalline and amorphous regions of cellulose respectively. Previous work has shown that CBM2a is a Type A CBM which binds to cellulose through a flat binding face incorporating a planar arrangement of hydrophobic aromatic residues [68,69]. This CBM has been shown by several researchers to be specific for crystalline cellulose [61,70,71]. Competitive binding experiments on PASC have been used to demonstrate that CBM2a has little binding site overlap with a Type B CBM (CBM4-1) known to exclusively recognize the amorphous regions of cellulose while showing no affinity for crystalline regions [61,71]. As CBM2a is thought to interact with 2–3 chains in the ordered crystal lattice [70], in the work reported here, we have defined a crystalline region as one with 2–3 adjacent crystalline cellulose chains. In contrast, the amorphous-cellulose binding CBM used in this work, CBM44, is a Type B CBM with a binding site comprised of a narrow groove lined with hydrophobic aromatic residues [72]. This groove confers binding specificity to free polysaccharide chains, such as those present in amorphous cellulose, but does not enable binding to the tightly packed chains found within crystalline cellulose [72].

To try to progressively disrupt cotton fibres in order to assess the ability of CBM adsorption to quantify cellulose accessibility a range of phosphoric acid concentrations was used. As the concentration of phosphoric acid was increased, an increase in the degree of disruption of the sample, including the splitting, delaminating and roughening of the fibres was apparent (Figure 2). An increase in disruption generally correlated with an increase in binding of both the amorphous-binding and crystalline-binding CBMs up to a concentration of 77% acid treatment (Figure 3). This increase in the combined binding of both CBMs provided a good indication that the overall (crystalline and amorphous) cellulose accessibility had increased. Surprisingly, the use of increasingly harsh acid treatments resulted in only a relatively small increase in the amount of CBM2a bound to the substrate when compared to the increase in CBM44 binding. It had been anticipated that the increasing disruption of the fibres would result in the amorphous-binding CBM44 being bound more than the crystalline-binding CBM2a. However, as CBM2a recognizes only two to three adjacent chains as being ‘crystalline’, the observed relative increase in CBM2a binding over CBM44 binding as the acid concentration increased was likely due to the increased solvent exposure of small microcrystalline substructures within the acid-disrupted cotton fibres.

As the acid concentration was further increased from 77% to 78%, the SEM micrographs of the cotton showed that any residual fibre structure had been lost and that the substrate now had the form of amorphous cellulosic ‘mats’ (Figure 2). These SEM observations complemented the CBM adsorption results, where increasing the acid concentration from 77% to 78% resulted in a large increase in the amount of adsorbed CBM44, without significantly altering the adsorption of CBM2a (Figure 3). It was apparent that the specificity of CBM adsorption was distinct enough that changes in the surface morphology of the cellulosic substrates could be readily differentiated.

**Figure 4:**
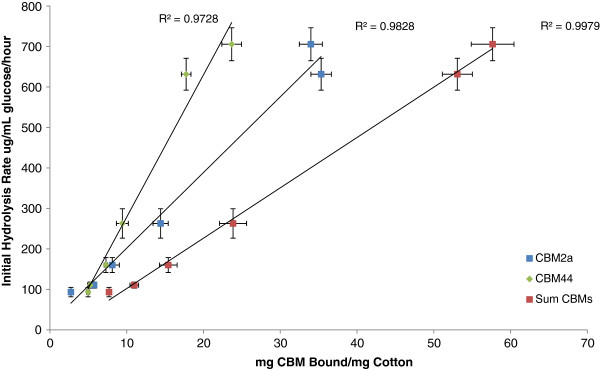
**Initial hydrolysis rate vs adsorption of CBMs.** Initial hydrolysis rate (calculated after 30 minutes of hydrolysis) of acid-disrupted cotton fibres increases with increasing CBM adsorption. Each data point represents a cotton fibre sample treated with a different concentration of *o-*phosphoric acid and hydrolyzed with the same enzyme loading. Experiments were run in triplicate and error bars represent one standard deviation from the mean.

After determining that CBM adsorption could be used to quantify acid-induced changes in cellulose accessibility, we attempted to correlate the degree of substrate disruption (quantified by CBM adsorption) with enzymatic hydrolyzability. Each of the phosphoric-acid disrupted cotton fibre samples was hydrolyzed using a commercial cellulase mixture (30 filter paper units/g cellulose of Celluclast 1.5 L, supplemented with 15 cellobiase units/g cellulose of Beta-glucosidase (Novozym 188, Novozymes A/S, Bagsværd, Denmark)). The initial hydrolysis rate (defined here as the hydrolysis rate over the first 30 minutes of the reaction) was plotted against the adsorption of each individual CBM, as well as the sum of their adsorptions (Figure 4). The adsorption of each individual CBM, and particularly their summed adsorptions, was found to correlate well with the enhanced enzymatic hydrolyzability of the cotton fibres. The steeper slope of the curve for CBM44 when compared to CBM2a seemed to indicate that, at least for the initial stages of hydrolysis, enzymatic hydrolysis rates were influenced more by the amount of accessible amorphous cellulose than they were by the amount of accessible crystalline cellulose.

Interestingly, although the hydrolyzability and CBM44 adsorption increased with every incremental increase in phosphoric acid concentration, this was not the case for CBM2a. As the acid concentration was increased from 77% to 78%, the adsorption of CBM2a to the substrate was not significantly affected, even as the hydrolyzability continued to increase. This suggested that any attempts to correlate changes in substrate accessibility to hydrolyzability using a specific mono-component cellulase may be problematic, as some cellulases contain CBMs specific for crystalline cellulose and might therefore underestimate the accessibility of the highly amorphous regions of the substrate.

This seemed to indicate that substructure specific CBMs could be used to quantify acid-induced changes in cellulose accessibility and that increases in accessibility (as determined by CBM adsorption) can provide a good predictor of initial rates of enzymatic hydrolysis.

### Quantification of Swollenin-induced increases in cellulose accessibility

**Figure 5:**
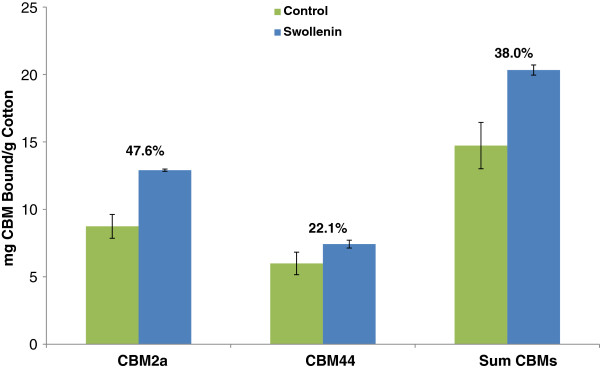
**Adsorption of CBMs to Swollenin-treated cotton fibres.** Swollenin-induced changes in the accessibility and surface morphology of mercerized cotton fibres quantified using CBM adsorption. The % values represent the % increase in adsorption of the CBMs after Swollenin treatment. A BSA negative protein control was found to have no significant effect on the extent of binding of either CBM. At least three replicates were performed for each sample. Error bars represent one standard deviation from the mean.

As Swollenin had previously been shown to disrupt mercerized cotton fibres [16], we next tried to quantify any changes in cellulose accessibility and surface morphology of mercerized cotton fibres treated with Swollenin by looking at the degree of adsorption of substructure-specific CBMs to the treated fibres. Although mercerization is known to cause a significant reduction in the crystallinity of cellulosic substrates, mercerized cellulose has been shown to retain some adsorptive capacity for crystalline binding CBMs [73]. After incubation with Swollenin, binding of both the crystalline and amorphous-specific CBMs to the mercerized cotton fibres increased (Figure 5).

The increase in binding was more pronounced forCBM2a than for CBM44 after Swollenin treatment, indicating that the increase in accessibility was not simply due to Swollenin-mediated decrystallization of the cellulose at the microfibril surface. This suggested that Swollenin might act by promoting the delamination or fibrillation of the substrate, or by promoting the “splitting” of microfibrils, thereby exposing new crystalline regions of cellulose to the CBMs.

**Figure 6:**
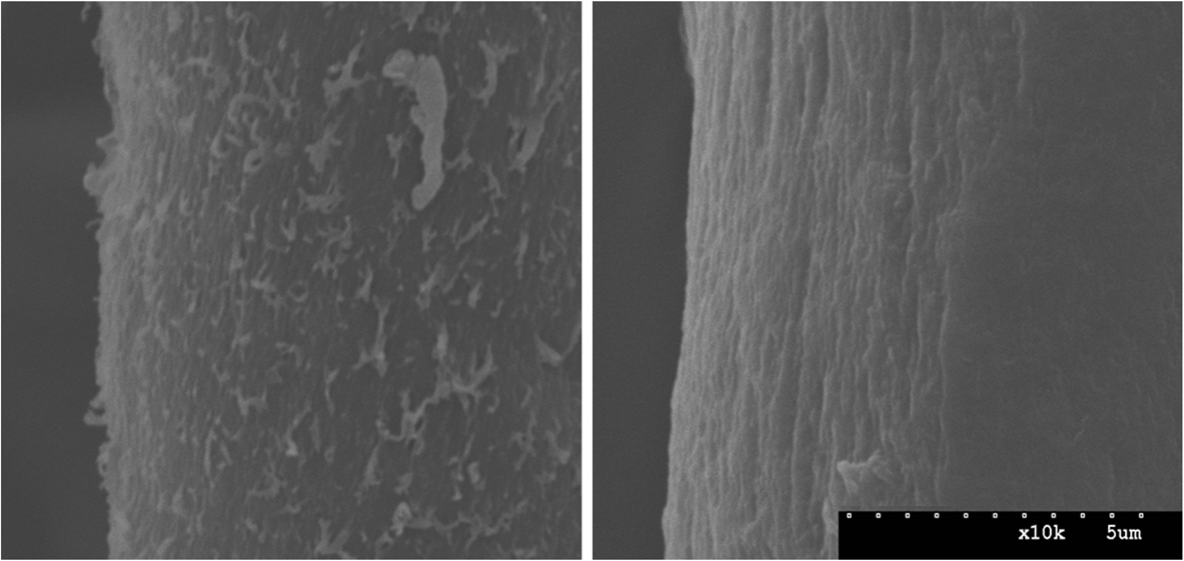
**Effect of Swollenin on mercerized cotton fibres imaged by SEM.** The surface of the control fibre (Left) appears roughened due to the mercerization treatment. The rough features on the surface of the mercerized cotton fibres appear to have been sloughed off by the action of Swollenin (Right). Images are of representative fibres for the indicated treatment, and are at 10000x magnification.

Subsequent SEM micrographs of Swollenin-treated mercerized cotton fibres indicated that Swollenin treatments resulted in a smoothing of the roughened patches produced during mercerization (Figure 6). This smoothing effect was in contrast to the buffer- and BSA-treated mercerized cotton fibres, which retained their roughened surface. After Swollenin treatment, the roughened patches at the surface of the fibres appeared to have been sloughed off, revealing the smooth, well ordered surface of the underlying cotton fibre. The observed increase in turbidity in the supernatant after Swollenin treatment (data not shown) was also indicative of the release of small particles into solution. It is possible that the roughened patches at the surface of the mercerized cotton fibres will contain a higher proportion of amorphous cellulose than the underlying fibre, as these protruding rough regions were more exposed to the NaOH used for mercerization. This treatment has been shown to promote the conversion of crystalline cellulose I into amorphous cellulose and crystalline cellulose II [74]. It is possible that the release of these roughened particles from the surface of the fibre resulted in an increase in both the amount of exposed amorphous cellulose (primarily on the released particles) and the amount of exposed crystalline cellulose (primarily on the newly exposed surface of the underlying cotton fibre). However, it should be noted that the small roughened particles that are released from the surface of the cotton fibres appear to be approximately 100 nm in the shorter direction, and up to 1000 nm in the longer direction (estimated from Figure 6). Since the cellulosic cores of cotton microfibrils have diameters of only 3–5 nm and lengths of 100 s to 1000 s of nm [5,48,49], it is possible that the small, roughened particles released from the surface of the cotton fibres still contained significant amounts of crystalline cellulose.

Although it was not evident by which specific mechanism the Swollenin resulted in this “smoothing” effect, it is possible that Swollenin acts in a similar manner to the Expansin family of proteins, which have been shown to weaken plant cell walls through disruption of the hydrogen bonding network between plant cell wall polymers [39]. If Swollenin disrupts hydrogen bonding in a similar mode to Expansins this might also explain how Swollenin appears to both disrupt the cell wall structure of Whatman filter paper No. 1 fibres and result in the swelling of cotton fibres [16,17].

These results indicated that substructure-specific CBMs could not only be used to track changes in cellulose accessibility after harsh acid treatments, but could also be used to track changes in surface morphology after the milder, Swollenin-induced, amorphogenesis. This technique has several advantages over current alternatives as it provided a direct, quantitative method able to consolidate changes in multiple substrate characteristics. Specifically, changes in the amounts of accessible amorphous cellulose, accessible crystalline cellulose and the total (amorphous and crystalline) accessible cellulose can all be quantified. It was also apparent that this method could help better indicate the mode of action of non-hydrolytic, disruptive/amorphogenesis-inducing proteins and has potential to yield novel insights into the mechanisms of glycosyl hydrolases and the other accessory enzymes involved in lignocellulose deconstruction. Additionally, this technique has the potential to facilitate comparisons of the disruptive capabilities of various non-hydrolytic proteins which might promote an increase in cellulose accessibility to the more traditional, hydrolytic components of the cellulase enzyme mixture.

It is also possible that CBMs with specificities for certain hemicelluloses, such as xylan or mannan, might be able to be used to track changes in the accessibility of these polymers during pretreatment, amorphogenesis and hydrolysis of softwoods, hardwoods and agricultural residues. In previous work, Filonova *et al.*, (2007) demonstrated the use of fluorescently-tagged mannan-specific CBM’s to quantify the accessibility of mannan in wood tissues and pulp, after applying a protein-based lignin-blocking technique to prevent non-specific adsorption of the CBMs to lignin [75]. The use of CBM-specific antibodies, or conjugation of CBMs to distinct fluorophores, have been used to provide direct visualization of the locations of the different polymers or substructures at the substrate surface [75,76]. Thus, by utilizing a suite of different CBMs with specificities for a range of structural features of the substrate, it might be possible to track changes in the morphology of the substrate during pretreatment and hydrolysis while better quantifying the role that enzyme access to the cellulose plays in limiting the rate and extent of enzymatic hydrolysis.

## Conclusions

Previous attempts to try and quantify the “cellulose swelling/delamination” or the “amorphogenesis step” of cellulose hydrolysis have tended to make use of a suite of complementary qualitative and semi-quantitative techniques. While these techniques have provided some useful information regarding the effects of amorphogenesis-inducing proteins on (ligno) cellulosic substrates, they have typically provided little insight into the mode of action of these proteins. A novel technique, using non-hydrolytic substructure-specific CBMs capable of quantitatively measuring changes in cellulose accessibility and surface morphology was successfully used to track changes in the cellulose during Swollenin-induced amorphogenesis. This novel method provided useful insights into how proteins such as Swollenin might increase cellulose accessibility by non-hydrolytic mechanisms such as the swelling or delamination of the cellulose substructures.

## Methods

### Proteins

CBM44 from *Clostridium thermocellum *was purchased from NZYTech (Lisbon, Portugal, CR0049). CBM2a from *Cellulomonas fimi* was provided by Dr. Douglas Kilburn from the University of British Columbia, Canada, after recombinant expression and purification from *E. coli*. Recombinant Swollenin (Swo1) was generously provided by VTT Technical Research Centre, Finland. Briefly, the Swollenin was expressed in *Trichoderma reesei* as a histidine-tagged protein and purified in two chromatographic steps.

### Phosphoric acid-induced amorphogenesis

Phosphoric acid-disrupted cotton fibres were prepared following a protocol similar to that described by Zhang *et al.* (2006) [67]. Briefly, ice-cold *o*-phosphoric acid (Fisher Scientific, Canada, A242) solutions were produced at various concentrations and 14.5 mL was added to 50 mL centrifuge tubes containing 0.2 g cotton fibres (Sigma-Aldrich, St. Louis MO, USA, C6663) pre-wetted with 0.5 mL nanopure water to give final *o*-phosphoric acid concentrations of 0–78% w/w. Samples were incubated for one hour on ice with occasional mixing. Ice-cold nanopure water (35 mL) was slowly added to each sample, followed by centrifugation at 10,000 g for 15 minutes. The fibres were resuspended in 50 mL nanopure water and washed a further 4 times with 50 mL nanopure water, followed by one wash with 50 mL 20 mM Na_2_CO_3_ (Fisher Scientific, Pittsburg PA, USA, S263) and 2 subsequent washes in 50 mL nanopure water. The cotton fibres were then lyophilized overnight.

### Swollenin-induced amorphogenesis

Prior to Swollenin treatment, 200 mg cotton fibres (Sigma-Aldrich, St. Louis MO, USA, C6663) were mercerized in 50 mL 25% (w/w) ice-cold NaOH for 15 minutes. The mercerized fibres were washed thoroughly with nanopure water then lyophilized overnight. Dried cotton fibres (50 mg) were weighed into 2 mL screwcap tubes. Swollenin or BSA (10 μg/mg cotton in 50 mM sodium acetate buffer, pH 5), or buffer alone was added and the samples were incubated overnight at 50°C in a FinepcrCombi SV12 hybridization incubator at 30 rpm. Protein was removed from the samples by extensive washing with nanopure water. Samples were lyophilized overnight prior to CBM adsorption and microscopy studies.

### Turbidity measurements

Supernatants from the Swollenin- or control-treated mercerized cotton fibres were transferred to 1 mL plastic cuvettes, and the optical density at 600 nm was read on a Varian Cary 50 Bio Spectrophotometer. Sodium acetate buffer (50 mM, pH 5) was used as a blank. Samples were run in triplicate.

### Scanning electron microscopy

Lyophilized cotton fibres were mounted on aluminum SEM stubs using double sided tape and sputter-coated with 10 nm Au/Pd (80:20 mix) then imaged on a Hitachi S-2600 VP-SEM (Tokyo, Japan).

### CBM adsorption

CBM2a and CBM44 were made up to 500 μg/mL in 50 mM sodium acetate buffer, pH 5, and added to 5 mg of phosphoric acid-treated or Swollenin-treated cotton fibres to a final CBM concentration of 50 μg/mg cotton. Samples were incubated for 30 minutes at 20°C in FinepcrCombi SV12 hybridization incubator at 30 rpm then centrifuged at 16,000 g for 10 minutes in a benchtop centrifuge. The amount of CBM bound to the cotton was calculated by measuring the absorbance of the supernatant at 280 nm and determining the concentration of the residual CBM in the supernatant using the calculated molar extinction coefficients of 27,625 M^-1^ and 27,365 M^-1^ for CBM2a and CBM44, respectively [77]. The amount of CBM bound to the cotton was calculated by subtracting the amount of the residual CBM in the supernatant from the original amount of CBM added to the sample.

### Enzymatic Hydrolysis

Cotton samples were hydrolyzed using 5 mg substrate in 1 mL 50 mM sodium acetate buffer, pH 5, at 50°C for 30 minutes, with an enzyme loading of 30 filter paper units Celluclast (Novozyme, USA) per gram cotton and supplemental β-glucosidase (Novozymes 188, Novozymes,Bagsværd, Denmark) at 1:2 cellobiase units/filter paper unit. After hydrolysis, the enzymes were heat inactivated at 100°C for 10 minutes, samples were centrifuged at 16,000 g in a benchtop centrifuge for 10 minutes, and the glucose concentration of the supernatant was determined using the glucose oxidase assay [46,47].

## Abbreviations

CBM, Carbohydrate binding module; SEM, Scanning electron microscopy; BSA, Bovine serum albumin.

## Competing interests

The authors declare that they have no competing interests.

## Authors' contributions

KG designed and carried out the experiments, analyzed results and drafted the manuscript. VA and JNS coordinated the study, helped write and review the manuscript. All authors read and approved the final manuscript.
